# 1,1′-(2,2-Di­phenyl­ethene-1,1-di­yl)bis­(3,5-dimethyl-1*H*-pyrazol-2-ium) dichloride

**DOI:** 10.1107/S2414314626005687

**Published:** 2026-06-02

**Authors:** Anke Schwarzer, Uwe Böhme

**Affiliations:** a Institut für Organische Chemie, Technische Universität Bergakademie Freiberg, Leipziger Str. 29, 09599 Freiberg, Germany; b Institut für Anorganische Chemie, Technische Universität Bergakademie Freiberg, Leipziger Str. 29, 09599 Freiberg, Germany; Purdue University, USA

**Keywords:** crystal structure, alkene, pyrazole, dianion

## Abstract

The title compound, C_24_H_26_N_4_^2+^·2Cl^−^, crystallizes in the triclinic space group *P*1. The ethene derivative is substituted by two phenyl groups in 2-position and two 3,5-di­methyl­pyrazolinium units in 1-position, which makes this alkene into a dication. Two chloride ions act as counter-ions in the crystal structure and are linked *via* N—H⋯Cl and C—H⋯Cl inter­actions with the alkene. The crystal structure features a three-dimensional network stabilized by N—H⋯Cl, C—H⋯Cl and C—H⋯π inter­actions. The crystal structure contains disordered solvent mol­ecules, corresponding to 43 electrons in a void volume of 102 Å^3^, which could not be modeled satisfactorily. Reverse Fourier transform methods were used to account for the unknown pore content.

## Abstract 2

The title compound crystallizes in the triclinic space group *P*1. The ethene derivative is substituted by two phenyl groups in 2-position and two 3,5-di­methyl­pyrazolinium units in 1-position, which makes this alkene into a dication. Two chloride ions act as counter-ions in the crystal structure and are linked *via* N—H⋯Cl and C—H⋯Cl inter­actions with the alkene. The crystal structure features a three-dimensional network stabilized by N—H⋯Cl, C—H⋯Cl and C—H⋯π inter­actions.

## Graphical abstract

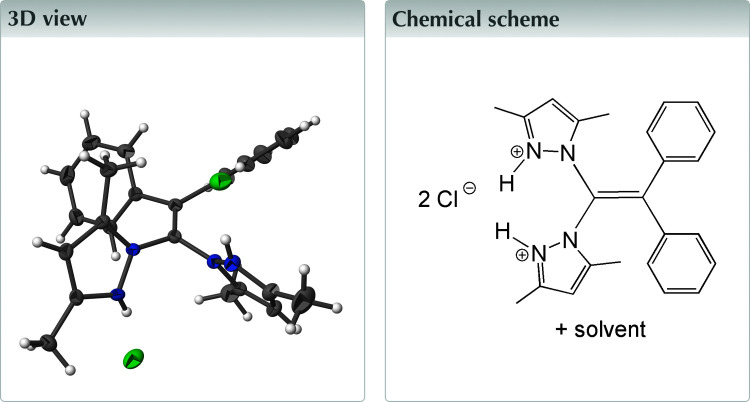

## Structure description

The title compound was obtained from the reaction of the heteroscorpionate ligand 2,2-bis­(3,5-di­methyl­pyrazol-1-yl)-1,1-di­phenyl­ethanol with europium trichloride in 1,2-di­meth­oxy­ethane solution. The formation of a heteroscorpionate complex was anti­cipated, as this ligand has previously been employed in the synthesis of various transition-metal, rare-earth-metal and group 14 element complexes (Hoffmann *et al.*, 2004 ▸, 2006 ▸; Tran & Carrano, 2007 ▸; Zhang *et al.*, 2010 ▸; Böhme *et al.*, 2019 ▸, 2025 ▸; Günther & Böhme, 2026 ▸). Unexpectedly, dehydration of the ligand occurred, resulting in the formation of the corresponding alkene.

**Figure 1:**
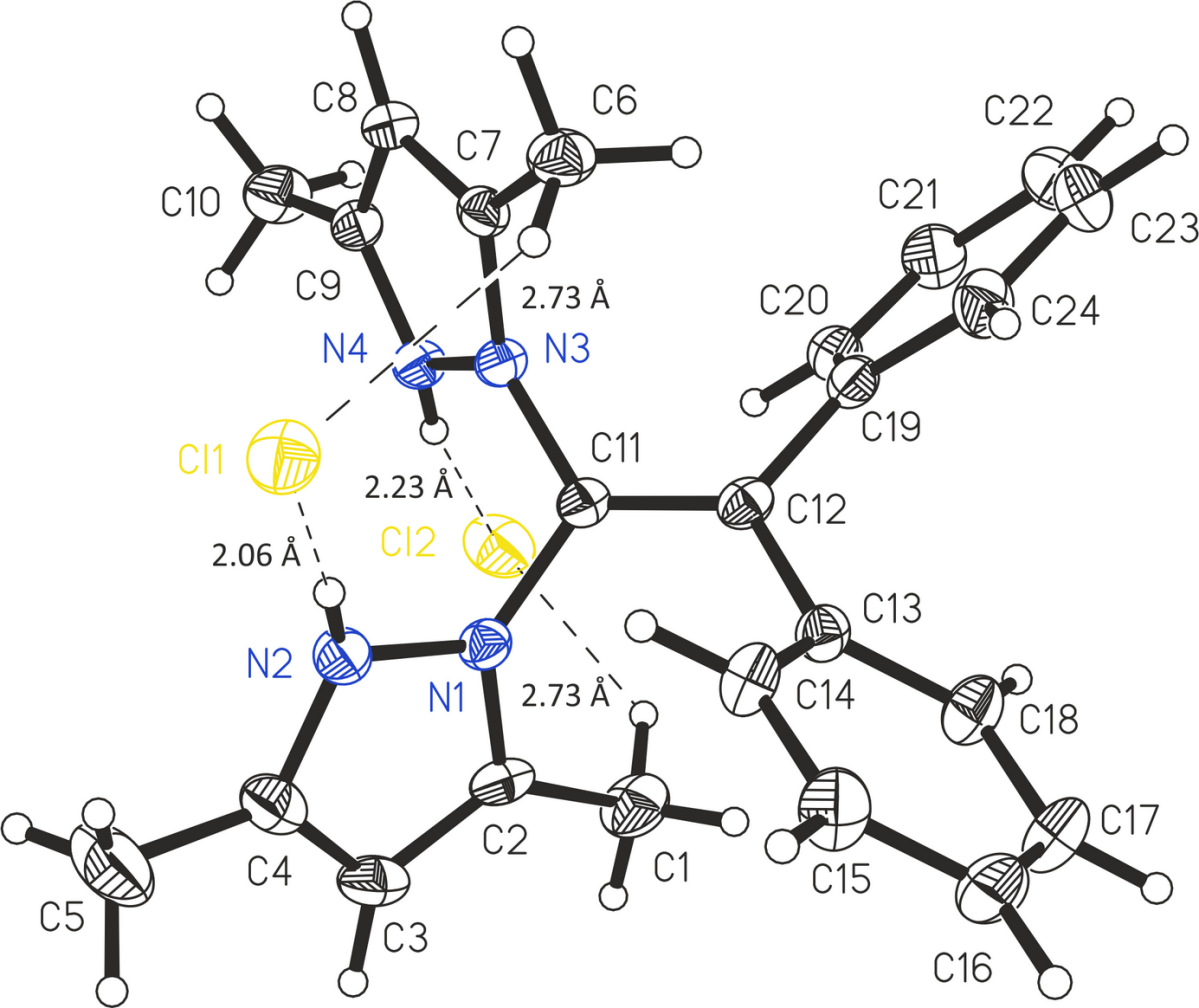
A view of the mol­ecular structure of the title compound, with the atom-labeling scheme. Displacement ellipsoids are drawn at the 50% probability level.

This alkene crystallizes in the triclinic space group *P*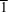. The asymmetric unit comprises one dicationic alkene species and two chloride counter-ions (Fig. 1 ▸). The crystal structure contains disordered solvent mol­ecules, which could not be modeled satisfactorily. Their contribution to the diffraction data was therefore accounted for using the SQUEEZE procedure in *PLATON* (Spek, 2020 ▸).

The ethene derivative is substituted by two di­methyl­pyrazolinium units at the 1-position and two phenyl groups at the 2-position. The asymmetric substitution raises the question of whether a push–pull alkene is present. Such systems are typically characterized by elongated C=C bond lengths (Ye *et al.*, 2010 ▸; Herbig & Böhme, 2023 ▸). However, the C11=C12 bond length in the title compound is 1.340 (3) Å (Table 1 ▸), which lies within the normal range for a C=C double bond and agrees well with the sum of the covalent radii for double-bonded carbon atoms (1.334 Å; Pauling, 1962 ▸). Therefore, it is to be concluded that the title compound is not a push–pull alkene.

The twisting of the C=C double bond is an additional feature to consider. Such twisting occurs in alkenes with steric crowding, which prevents planarity (Beck *et al.*, 1994 ▸; Bergmann *et al.*, 1953 ▸; Schollmeyer & Detert, 2022 ▸). The angle between the planes defined by atoms N1/C11/N3 and C13/C12/C19 is 23.4 (2)°, indicating moderate steric hindrance from the substituents in the title compound. The bond lengths C12—C13 [1.486 (2) Å] and C12—C19 [1.480 (3) Å] are slightly shorter than typical C—C single bonds (Pauling, 1962 ▸). Similarly, the N1—C11 [1.415 (2) Å] and N3—C11 [1.426 (2) Å] distances are somewhat shorter than expected for pure single bonds.

**Figure 2:**
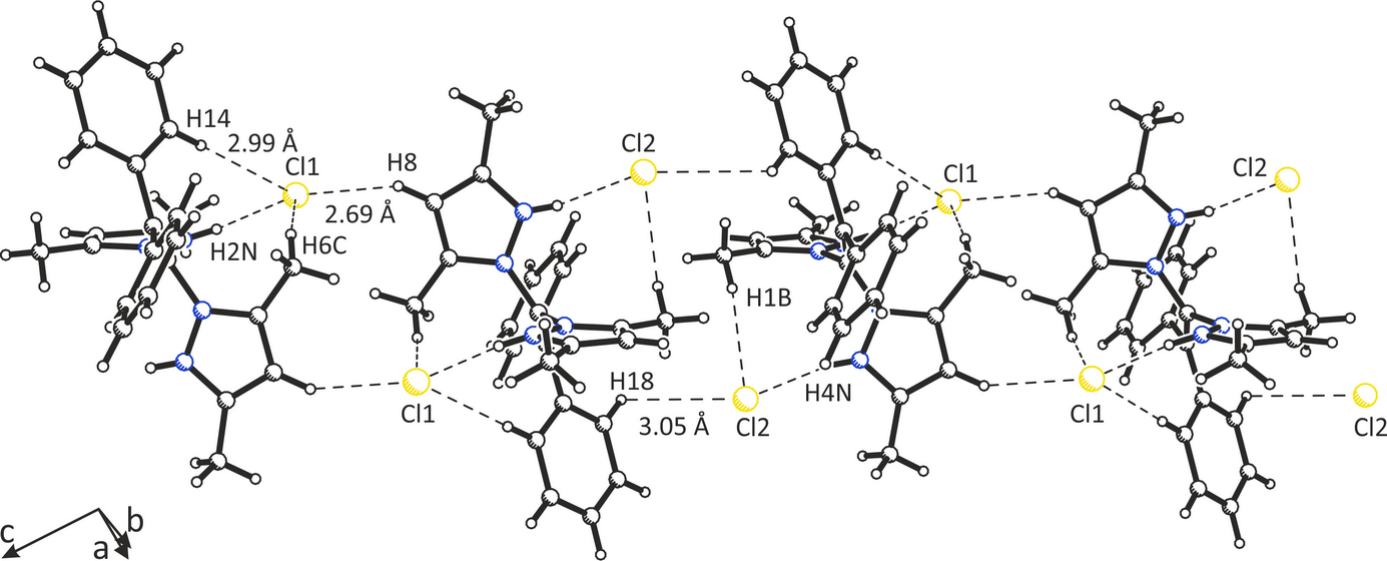
Partial packing diagram showing several N—H⋯Cl and C-H⋯Cl contacts forming a mol­ecular chain.

**Figure 3:**
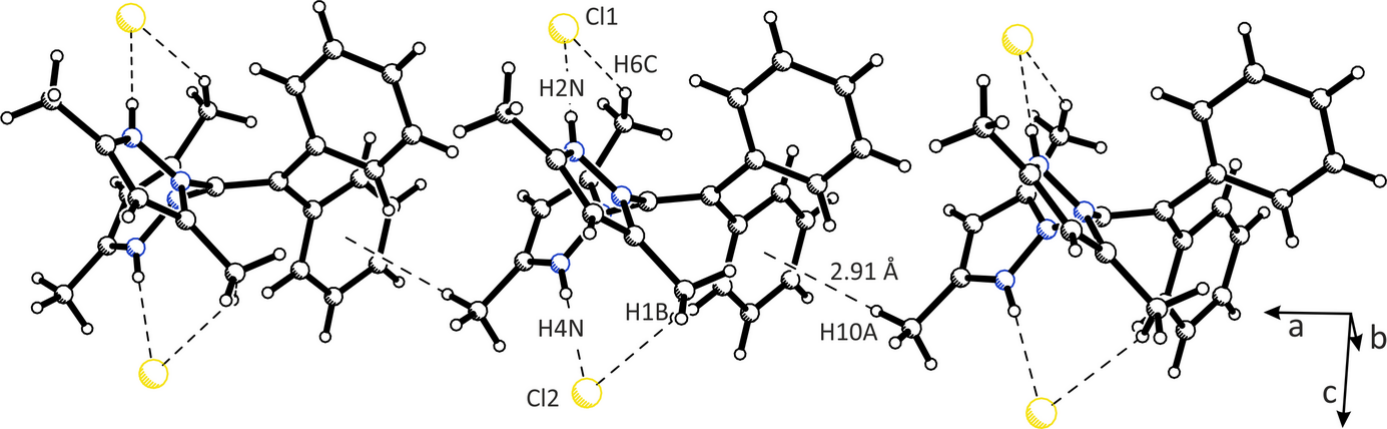
Partial packing diagram showing the C10—H10*A*⋯π inter­action connecting adjacent mol­ecules, forming a chain along the *a*-axis direction.

The chloride ions are linked to the di­methyl­pyrazolium units *via* short N—H⋯Cl hydrogen bonds (N2—H2*N*⋯Cl1 and N4—H4*N*⋯Cl2; Table 2 ▸). Two longer C—H⋯Cl inter­actions (C6—H6*C*⋯Cl1 and C1—H1*B*⋯Cl2) further consolidate the conformation of the di­methyl­pyrazolium units. These H⋯Cl inter­actions result in a mol­ecular chain combined with additional C8—H8⋯Cl1 (2.68 Å) and C18—H18⋯Cl2 (3.05 Å) inter­actions. This chain is shown in Fig. 2 ▸. The expansion by C23—H23⋯Cl1 generates a double layer of mol­ecules parallel to the crystallographic *ab* plane. These layers are connected along the *c*-axis direction by C10—H10*B*⋯Cl2 inter­actions, resulting in a three-dimensional network stabilized by both N—H⋯Cl and C—H⋯Cl inter­actions. Furthermore, one C10—H10*A*⋯π inter­action of 2.91 Å (Fig. 3 ▸) generates a mol­ecular chain presented in Fig. 3 ▸. This C—H⋯π contact also connects the described adjacent chains of mol­ecules generated by H⋯Cl contacts and completes the mol­ecular network of alkene mol­ecules and chloride ions.

A search of the Cambridge Structural Database (CSD, Version 6.01, November 2025 update; Groom *et al.*, 2016 ▸) revealed 32 structures containing a 1,1-bis­(pyrazol-1-yl)alkene motif. A notable related structure is tetra­kis­(1*H*-pyrazol-1-yl)ethene (CSD refcode HORWAQ; Takemasa & Nozaki, 2024 ▸). The C=C double bond length in this structure (1.344 Å) is nearly identical to that in the title compound. The C—N bond lengths in tetra­kis­(1*H*-pyrazol-1-yl)ethene are 1.40 Å, which is shorter than in the title compound, and the substituents at the C=C double bond generate a twist angle of 16.03°. Several structurally characterized aluminium complexes incorporating a 1,1-bis­(pyrazol-1-yl)alkene ligand have also been reported, typically formed from reactions of aluminium alkyls with scorpionate ligands (Castro-Osma *et al.*, 2013 ▸; Navarro *et al.*, 2020 ▸, 2023 ▸). Additionally, a series of 1,1-bis­(pyrazol-1-yl)alkenes was prepared *via* an NaOH-promoted reaction of 1,1-dihaloalkenes with 1*H*-azoles, although these compounds were not structurally characterized (Zhang *et al.*, 2018 ▸).

## Synthesis and crystallization

2,2-Bis(3,5-di­methyl­pyrazol-1-yl)-1,1-di­phenyl­ethanol (0.77 g, 1.99 mmol) and europium trichloride (0.52 g, 2.0 mmol) were dissolved separately in 1,2-di­meth­oxy­ethane (60 ml and 140 ml, respectively) under an argon atmosphere. The clear solutions were combined and left standing at room temperature. After six weeks, no visible reaction had occurred. The solvent was then reduced *in vacuo* to approximately one third of the original volume, and the resulting solution was stored at 8 °C. After two weeks, colourless flat prisms of the title compound suitable for X-ray diffraction analysis were obtained (m.p. = 174 °C). No yield could be determined because only a few crystals deposited on the wall of the Schlenk tube were isolated. NMR spectroscopic analysis of the batch product, which remained as an oily residue after evaporation, indicated the presence of a complex mixture of components. Further purification attempts were unsuccessful.

## Refinement

**Figure 4:**
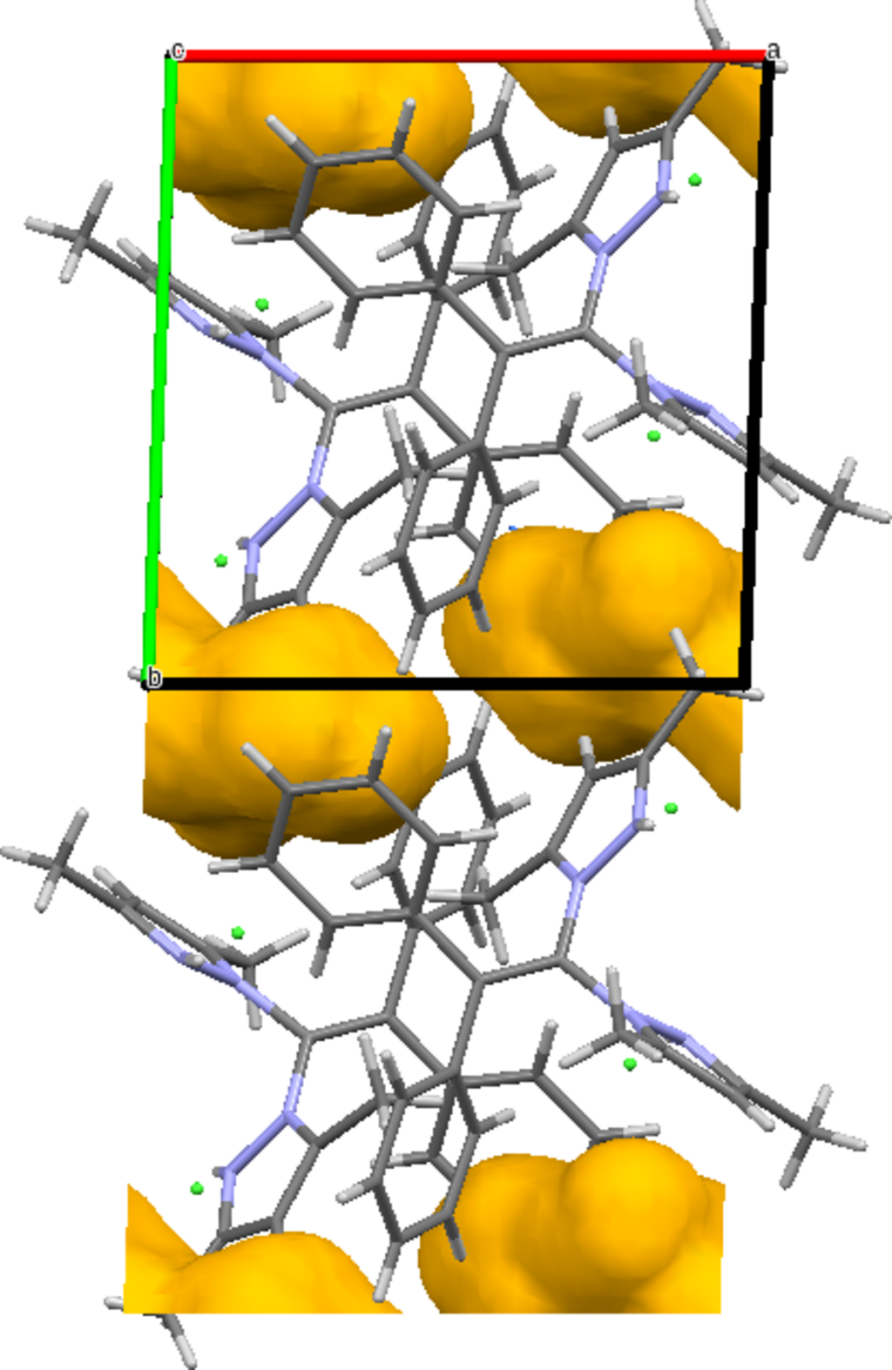
Brown–yellow areas show solvent-accessible voids in the unit cell of the title compound. View along crystallographic *c*-axis.

Crystal data, data collection, and structure refinement details are summarized in Table 3 ▸. Hydrogen atoms at nitro­gen atoms N2 and N4 were localized from residual electron-density maps and were freely refined. Hydrogen atoms bonded to C were positioned geometrically and allowed to ride on their parent atoms, with C—H = 0.95 Å for H(Ph), 0.95 for CH, and 0.98 Å for CH_3_. *U*_iso_(H) = *xU*_eq_(C), where *x* = 1.2 for H(Ph) and CH, and 1.5 for CH_3_. A pronounced residual electron-density peak (12.20 e^−^·Å^−3^) was observed at (0.7656, −0.0851, 0.5586). Attempts to model this density as either water or hydrogen chloride – including mixed site occupancy – did not yield satisfactory refinement results. Consequently, the contribution of the disordered solvent was accounted for using the SQUEEZE (Spek, 2015 ▸) procedure in *PLATON* (Spek, 2020 ▸). The solvent-accessible void volume was calculated to be 102 Å^3^ per unit cell (8.3% of the unit-cell volume), corresponding to 43 electrons. The voids in the unit cell are illustrated in Fig. 4 ▸.

## Supplementary Material

Crystal structure: contains datablock(s) I. DOI: 10.1107/S2414314626005687/zl4099sup1.cif

Structure factors: contains datablock(s) I. DOI: 10.1107/S2414314626005687/zl4099Isup2.hkl

Supporting information file. DOI: 10.1107/S2414314626005687/zl4099Isup3.cml

CCDC reference: 2557894

Additional supporting information:  crystallographic information; 3D view; checkCIF report

## Figures and Tables

**Table 1:** Selected geometric parameters (Å, °)

N1—C11	1.415 (2)	C12—C19	1.480 (3)
N3—C11	1.426 (2)	C12—C13	1.486 (2)
C11—C12	1.340 (3)		
			
C12—C11—N1	122.68 (15)	C11—C12—C19	121.22 (16)
C12—C11—N3	122.91 (16)	C11—C12—C13	120.77 (17)
N1—C11—N3	114.39 (15)	C19—C12—C13	118.01 (15)

**Table 2:** Hydrogen-bond geometry (Å, °) *Cg*4 is defined as the centre of gravity of the C19–C24 ring.

*D*—H⋯*A*	*D*—H	H⋯*A*	*D*⋯*A*	*D*—H⋯*A*
N2—H2*N*⋯Cl1	0.87 (2)	2.02 (2)	2.8853 (18)	172 (2)
N4—H4*N*⋯Cl2	0.78 (2)	2.24 (2)	3.0001 (17)	165 (2)
C1—H1*B*⋯Cl2	0.98	2.74	3.577 (2)	144
C6—H6*C*⋯Cl1	0.98	2.73	3.614 (2)	150
C8—H8⋯Cl1^i^	0.95	2.68	3.5352 (19)	149
C10—H10*A*⋯*Cg*4^ii^	0.98	2.91	3.871 (2)	168
C10—H10*B*⋯Cl2^iii^	0.98	2.69	3.577 (2)	151
C14—H14⋯Cl1	0.95	2.99	3.944 (2)	179
C18—H18⋯Cl2^iv^	0.95	3.05	3.553 (2)	115
C23—H23⋯Cl1^v^	0.95	2.74	3.602 (2)	151

Symmetry codes: (i) 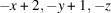; (ii) 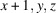; (iii) 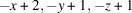; (iv) 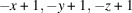; (v) 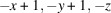.

**Table 3:** Experimental details

Crystal data	
Chemical formula	C_24_H_26_N_4_^2+^·2Cl^−^
*M*_r_	441.39
Crystal system, space group	Triclinic, *P* 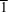
Temperature (K)	153
*a*, *b*, *c* (Å)	9.3202 (13), 10.0104 (16), 13.4409 (19)
α, β, γ (°)	101.063 (12), 90.783 (12), 92.021 (12)
*V* (Å^3^)	1229.7 (3)
*Z*	2
Radiation type	Mo *K*α
μ (mm^−1^)	0.28
Crystal size (mm)	0.12 × 0.09 × 0.07

Data collection	
Diffractometer	Stoe Stadivari
Absorption correction	Multi-scan (*LANA*; Koziskova *et al.*, 2016 ▸)
*T*_min_, *T*_max_	0.966, 0.980
No. of measured, independent and observed [*I* > 2σ(*I*)] reflections	18584, 6285, 4089
*R*_int_	0.027
(sin θ/λ)_max_ (Å^−1^)	0.703

Refinement	
*R*[*F*^2^ > 2σ(*F*^2^)], *wR*(*F*^2^), *S*	0.045, 0.127, 1.06
No. of reflections	6285
No. of parameters	281
H-atom treatment	H atoms treated by a mixture of independent and constrained refinement
Δρ_max_, Δρ_min_ (e Å^−3^)	0.40, −0.52

Computer programs: *X-AREAPilatus3_SV*, *Recipe*, *Integrate* and *LANA* (Stoe, 2023 ▸), *SHELXT2018/2* (Sheldrick, 2015*a* ▸), *SHELXL2019/2* (Sheldrick, 2015*b* ▸), *ORTEP-3 for Windows* and *WinGX* (Farrugia, 2012 ▸), *publCIF* (Westrip, 2010 ▸) and *ShelXle* (Hübschle *et al.*, 2011 ▸).

## full crystallographic data

### 1,1'-(2,2-Diphenylethene-1,1-diyl)bis(3,5-dimethyl-1H-pyrazol-2-ium) dichloride Crystal data

**Table:**

C_24_H_26_N_4_^2+^·2Cl^−^	*F*(000) = 464
*M**_r_* = 441.39	*D*_x_ = 1.192 Mg m^−^^3^
Triclinic, *P*1	Melting point: 447 K
*a* = 9.3202 (13) Å	Mo *K*α radiation, λ = 0.71073 Å
*b* = 10.0104 (16) Å	Cell parameters from 4634 reflections
*c* = 13.4409 (19) Å	θ = 2.6–26.9°
α = 101.063 (12)°	µ = 0.28 mm^−^^1^
β = 90.783 (12)°	*T* = 153 K
γ = 92.021 (12)°	Piece, colorless
*V* = 1229.7 (3) Å^3^	0.12 × 0.09 × 0.07 mm
*Z* = 2	

### 1,1'-(2,2-Diphenylethene-1,1-diyl)bis(3,5-dimethyl-1H-pyrazol-2-ium) dichloride Data collection

**Table:**

Stoe Stadivari diffractometer	6285 independent reflections
Radiation source: Primux 50 Mo	4089 reflections with *I* > 2σ(*I*)
Graded multilayer mirror monochromator	*R*_int_ = 0.027
Detector resolution: 5.81 pixels mm^-1^	θ_max_ = 30.0°, θ_min_ = 2.1°
rotation method, ω scans	*h* = −12→11
Absorption correction: multi-scan (*LANA*; Koziskova *et al.*, 2016)	*k* = −13→13
*T*_min_ = 0.966, *T*_max_ = 0.980	*l* = −18→18
18584 measured reflections	

### 1,1'-(2,2-Diphenylethene-1,1-diyl)bis(3,5-dimethyl-1H-pyrazol-2-ium) dichloride Refinement

**Table:**

Refinement on *F*^2^	Primary atom site location: structure-invariant direct methods
Least-squares matrix: full	Hydrogen site location: mixed
*R*[*F*^2^ > 2σ(*F*^2^)] = 0.045	H atoms treated by a mixture of independent and constrained refinement
*wR*(*F*^2^) = 0.127	*w* = 1/[σ^2^(*F*_o_^2^) + (0.0662*P*)^2^] where *P* = (*F*_o_^2^ + 2*F*_c_^2^)/3
*S* = 1.06	(Δ/σ)_max_ = 0.001
6285 reflections	Δρ_max_ = 0.40 e Å^−^^3^
281 parameters	Δρ_min_ = −0.52 e Å^−^^3^
0 restraints	

### 1,1'-(2,2-Diphenylethene-1,1-diyl)bis(3,5-dimethyl-1H-pyrazol-2-ium) dichloride Special details

**Table:**

Geometry. All esds (except the esd in the dihedral angle between two l.s. planes) are estimated using the full covariance matrix. The cell esds are taken into account individually in the estimation of esds in distances, angles and torsion angles; correlations between esds in cell parameters are only used when they are defined by crystal symmetry. An approximate (isotropic) treatment of cell esds is used for estimating esds involving l.s. planes.

### 1,1'-(2,2-Diphenylethene-1,1-diyl)bis(3,5-dimethyl-1H-pyrazol-2-ium) dichloride Fractional atomic coordinates and isotropic or equivalent isotropic displacement parameters (Å^2^)

**Table:**

	*x*	*y*	*z*	*U*_iso_*/*U*_eq_	
Cl1	0.88462 (5)	0.19683 (5)	0.00919 (4)	0.02918 (13)	
Cl2	0.83278 (6)	0.60437 (6)	0.53940 (4)	0.03838 (15)	
N1	0.73698 (15)	0.31350 (16)	0.27173 (11)	0.0192 (3)	
N2	0.83054 (16)	0.22323 (16)	0.22280 (13)	0.0222 (3)	
H2N	0.849 (2)	0.224 (2)	0.1597 (19)	0.033*	
N3	0.83160 (15)	0.52610 (15)	0.24065 (11)	0.0180 (3)	
N4	0.92219 (16)	0.56469 (16)	0.32245 (12)	0.0191 (3)	
H4N	0.897 (2)	0.560 (2)	0.3767 (18)	0.029*	
C1	0.5808 (2)	0.3404 (2)	0.42402 (16)	0.0330 (5)	
H1A	0.487201	0.335799	0.389100	0.049*	
H1B	0.613477	0.435892	0.445254	0.049*	
H1C	0.571857	0.298161	0.483767	0.049*	
C2	0.68647 (19)	0.26671 (19)	0.35419 (14)	0.0225 (4)	
C3	0.7502 (2)	0.1459 (2)	0.35491 (16)	0.0278 (4)	
H3	0.736923	0.089994	0.403984	0.033*	
C4	0.8376 (2)	0.1195 (2)	0.27114 (16)	0.0284 (4)	
C5	0.9246 (3)	−0.0003 (2)	0.2321 (2)	0.0489 (6)	
H5A	0.925229	−0.015808	0.157827	0.073*	
H5B	0.882779	−0.080966	0.253707	0.073*	
H5C	1.023240	0.016997	0.258886	0.073*	
C6	0.8150 (2)	0.5562 (2)	0.06021 (14)	0.0261 (4)	
H6A	0.878099	0.592523	0.013189	0.039*	
H6B	0.723886	0.602647	0.064436	0.039*	
H6C	0.796799	0.458305	0.035681	0.039*	
C7	0.88503 (18)	0.57896 (18)	0.16187 (13)	0.0192 (4)	
C8	1.00871 (19)	0.65338 (19)	0.19719 (14)	0.0221 (4)	
H8	1.069609	0.701880	0.159186	0.027*	
C9	1.02837 (18)	0.64475 (19)	0.29835 (15)	0.0210 (4)	
C10	1.1393 (2)	0.7105 (2)	0.37447 (16)	0.0313 (5)	
H10A	1.225357	0.734217	0.339528	0.047*	
H10B	1.163796	0.647369	0.418913	0.047*	
H10C	1.101654	0.793350	0.415111	0.047*	
C11	0.70831 (18)	0.43791 (18)	0.24155 (13)	0.0183 (4)	
C12	0.57581 (18)	0.47095 (19)	0.21768 (13)	0.0189 (4)	
C13	0.46216 (18)	0.36357 (19)	0.18285 (14)	0.0199 (4)	
C14	0.49132 (19)	0.2494 (2)	0.11020 (15)	0.0255 (4)	
H14	0.585742	0.237258	0.085165	0.031*	
C15	0.3821 (2)	0.1527 (2)	0.07406 (17)	0.0316 (5)	
H15	0.402186	0.075519	0.023782	0.038*	
C16	0.2452 (2)	0.1686 (2)	0.11088 (17)	0.0321 (5)	
H16	0.171114	0.102350	0.086272	0.038*	
C17	0.2162 (2)	0.2808 (2)	0.18346 (17)	0.0329 (5)	
H17	0.122130	0.291126	0.209492	0.039*	
C18	0.32296 (19)	0.3788 (2)	0.21889 (15)	0.0262 (4)	
H18	0.301248	0.456787	0.267968	0.031*	
C19	0.53795 (18)	0.61484 (19)	0.22543 (14)	0.0209 (4)	
C20	0.57768 (19)	0.7103 (2)	0.31193 (15)	0.0248 (4)	
H20	0.634446	0.683972	0.363624	0.030*	
C21	0.5351 (2)	0.8434 (2)	0.32315 (17)	0.0316 (5)	
H21	0.561091	0.907354	0.383047	0.038*	
C22	0.4546 (2)	0.8838 (2)	0.24741 (18)	0.0359 (5)	
H22	0.427099	0.975557	0.254793	0.043*	
C23	0.4144 (2)	0.7898 (2)	0.16077 (17)	0.0331 (5)	
H23	0.359779	0.817287	0.108523	0.040*	
C24	0.4541 (2)	0.6558 (2)	0.15055 (16)	0.0277 (4)	
H24	0.423985	0.591120	0.092017	0.033*	

### 1,1'-(2,2-Diphenylethene-1,1-diyl)bis(3,5-dimethyl-1H-pyrazol-2-ium) dichloride Atomic displacement parameters (Å^2^)

**Table:**

	*U*^11^	*U*^22^	*U*^33^	*U*^12^	*U*^13^	*U*^23^
Cl1	0.0295 (3)	0.0368 (3)	0.0221 (2)	0.0030 (2)	0.00231 (19)	0.0076 (2)
Cl2	0.0541 (3)	0.0374 (3)	0.0260 (3)	0.0111 (2)	0.0127 (2)	0.0096 (2)
N1	0.0182 (7)	0.0219 (8)	0.0181 (8)	0.0002 (6)	0.0024 (6)	0.0058 (6)
N2	0.0237 (8)	0.0227 (9)	0.0213 (8)	0.0037 (6)	0.0037 (7)	0.0059 (7)
N3	0.0166 (7)	0.0217 (8)	0.0164 (7)	−0.0025 (6)	−0.0020 (6)	0.0062 (6)
N4	0.0196 (8)	0.0233 (8)	0.0153 (8)	−0.0019 (6)	−0.0023 (6)	0.0062 (7)
C1	0.0315 (11)	0.0422 (13)	0.0262 (11)	−0.0008 (9)	0.0090 (9)	0.0091 (10)
C2	0.0227 (9)	0.0260 (10)	0.0195 (9)	−0.0083 (8)	−0.0009 (7)	0.0078 (8)
C3	0.0335 (11)	0.0274 (11)	0.0249 (10)	−0.0050 (8)	−0.0001 (8)	0.0124 (9)
C4	0.0327 (11)	0.0246 (11)	0.0300 (11)	0.0026 (8)	−0.0022 (9)	0.0103 (9)
C5	0.0671 (17)	0.0325 (13)	0.0513 (16)	0.0200 (12)	0.0129 (13)	0.0147 (12)
C6	0.0286 (10)	0.0307 (11)	0.0206 (10)	−0.0025 (8)	−0.0004 (8)	0.0095 (8)
C7	0.0209 (9)	0.0206 (9)	0.0173 (9)	0.0030 (7)	0.0019 (7)	0.0061 (7)
C8	0.0209 (9)	0.0212 (10)	0.0254 (10)	−0.0021 (7)	0.0024 (8)	0.0077 (8)
C9	0.0184 (9)	0.0189 (9)	0.0258 (10)	−0.0015 (7)	−0.0005 (7)	0.0049 (8)
C10	0.0320 (11)	0.0303 (12)	0.0312 (11)	−0.0088 (9)	−0.0093 (9)	0.0070 (9)
C11	0.0179 (8)	0.0199 (9)	0.0174 (9)	−0.0013 (7)	0.0009 (7)	0.0044 (7)
C12	0.0189 (9)	0.0228 (10)	0.0151 (9)	−0.0014 (7)	0.0017 (7)	0.0040 (7)
C13	0.0178 (8)	0.0228 (10)	0.0192 (9)	−0.0010 (7)	−0.0027 (7)	0.0049 (8)
C14	0.0182 (9)	0.0309 (11)	0.0255 (10)	−0.0002 (8)	0.0007 (8)	0.0013 (9)
C15	0.0287 (10)	0.0263 (11)	0.0353 (12)	0.0005 (8)	−0.0047 (9)	−0.0048 (9)
C16	0.0249 (10)	0.0319 (12)	0.0380 (12)	−0.0093 (9)	−0.0068 (9)	0.0056 (10)
C17	0.0184 (9)	0.0405 (13)	0.0380 (12)	−0.0052 (8)	0.0036 (9)	0.0041 (10)
C18	0.0208 (9)	0.0277 (11)	0.0271 (10)	−0.0007 (8)	0.0031 (8)	−0.0019 (8)
C19	0.0166 (9)	0.0237 (10)	0.0226 (10)	−0.0013 (7)	0.0003 (7)	0.0053 (8)
C20	0.0215 (9)	0.0251 (10)	0.0278 (10)	−0.0016 (8)	−0.0035 (8)	0.0060 (8)
C21	0.0325 (11)	0.0241 (11)	0.0361 (12)	−0.0019 (8)	−0.0036 (9)	0.0015 (9)
C22	0.0355 (12)	0.0243 (11)	0.0496 (14)	0.0033 (9)	0.0023 (10)	0.0110 (10)
C23	0.0283 (11)	0.0385 (13)	0.0360 (12)	0.0043 (9)	−0.0052 (9)	0.0163 (10)
C24	0.0252 (10)	0.0328 (11)	0.0250 (10)	0.0010 (8)	−0.0039 (8)	0.0056 (9)

### 1,1'-(2,2-Diphenylethene-1,1-diyl)bis(3,5-dimethyl-1H-pyrazol-2-ium) dichloride Geometric parameters (Å, º)

**Table:**

N1—N2	1.361 (2)	C10—H10A	0.9800
N1—C2	1.367 (2)	C10—H10B	0.9800
N1—C11	1.415 (2)	C10—H10C	0.9800
N2—C4	1.329 (2)	C11—C12	1.340 (3)
N2—H2N	0.87 (2)	C12—C19	1.480 (3)
N3—N4	1.364 (2)	C12—C13	1.486 (2)
N3—C7	1.364 (2)	C13—C14	1.391 (3)
N3—C11	1.426 (2)	C13—C18	1.394 (3)
N4—C9	1.334 (2)	C14—C15	1.395 (3)
N4—H4N	0.78 (2)	C14—H14	0.9500
C1—C2	1.486 (3)	C15—C16	1.379 (3)
C1—H1A	0.9800	C15—H15	0.9500
C1—H1B	0.9800	C16—C17	1.376 (3)
C1—H1C	0.9800	C16—H16	0.9500
C2—C3	1.367 (3)	C17—C18	1.385 (3)
C3—C4	1.389 (3)	C17—H17	0.9500
C3—H3	0.9500	C18—H18	0.9500
C4—C5	1.487 (3)	C19—C20	1.393 (3)
C5—H5A	0.9800	C19—C24	1.397 (3)
C5—H5B	0.9800	C20—C21	1.385 (3)
C5—H5C	0.9800	C20—H20	0.9500
C6—C7	1.480 (3)	C21—C22	1.386 (3)
C6—H6A	0.9800	C21—H21	0.9500
C6—H6B	0.9800	C22—C23	1.387 (3)
C6—H6C	0.9800	C22—H22	0.9500
C7—C8	1.378 (2)	C23—C24	1.386 (3)
C8—C9	1.389 (3)	C23—H23	0.9500
C8—H8	0.9500	C24—H24	0.9500
C9—C10	1.488 (3)		
			
N2—N1—C2	108.86 (15)	C9—C10—H10A	109.5
N2—N1—C11	123.02 (14)	C9—C10—H10B	109.5
C2—N1—C11	128.02 (16)	H10A—C10—H10B	109.5
C4—N2—N1	108.59 (15)	C9—C10—H10C	109.5
C4—N2—H2N	128.3 (15)	H10A—C10—H10C	109.5
N1—N2—H2N	118.5 (15)	H10B—C10—H10C	109.5
N4—N3—C7	108.51 (13)	C12—C11—N1	122.68 (15)
N4—N3—C11	122.81 (14)	C12—C11—N3	122.91 (16)
C7—N3—C11	128.57 (15)	N1—C11—N3	114.39 (15)
C9—N4—N3	109.11 (15)	C11—C12—C19	121.22 (16)
C9—N4—H4N	127.2 (16)	C11—C12—C13	120.77 (17)
N3—N4—H4N	120.3 (16)	C19—C12—C13	118.01 (15)
C2—C1—H1A	109.5	C14—C13—C18	118.93 (17)
C2—C1—H1B	109.5	C14—C13—C12	120.49 (16)
H1A—C1—H1B	109.5	C18—C13—C12	120.51 (17)
C2—C1—H1C	109.5	C13—C14—C15	120.08 (17)
H1A—C1—H1C	109.5	C13—C14—H14	120.0
H1B—C1—H1C	109.5	C15—C14—H14	120.0
N1—C2—C3	106.69 (17)	C16—C15—C14	120.4 (2)
N1—C2—C1	122.91 (18)	C16—C15—H15	119.8
C3—C2—C1	130.39 (18)	C14—C15—H15	119.8
C2—C3—C4	107.81 (17)	C17—C16—C15	119.70 (18)
C2—C3—H3	126.1	C17—C16—H16	120.2
C4—C3—H3	126.1	C15—C16—H16	120.2
N2—C4—C3	108.01 (18)	C16—C17—C18	120.59 (18)
N2—C4—C5	121.34 (18)	C16—C17—H17	119.7
C3—C4—C5	130.62 (19)	C18—C17—H17	119.7
C4—C5—H5A	109.5	C17—C18—C13	120.30 (19)
C4—C5—H5B	109.5	C17—C18—H18	119.8
H5A—C5—H5B	109.5	C13—C18—H18	119.8
C4—C5—H5C	109.5	C20—C19—C24	118.77 (18)
H5A—C5—H5C	109.5	C20—C19—C12	119.83 (17)
H5B—C5—H5C	109.5	C24—C19—C12	121.25 (18)
C7—C6—H6A	109.5	C21—C20—C19	120.49 (19)
C7—C6—H6B	109.5	C21—C20—H20	119.8
H6A—C6—H6B	109.5	C19—C20—H20	119.8
C7—C6—H6C	109.5	C20—C21—C22	120.3 (2)
H6A—C6—H6C	109.5	C20—C21—H21	119.9
H6B—C6—H6C	109.5	C22—C21—H21	119.9
N3—C7—C8	106.94 (15)	C21—C22—C23	119.9 (2)
N3—C7—C6	123.36 (15)	C21—C22—H22	120.1
C8—C7—C6	129.70 (16)	C23—C22—H22	120.1
C7—C8—C9	107.71 (15)	C24—C23—C22	119.9 (2)
C7—C8—H8	126.1	C24—C23—H23	120.1
C9—C8—H8	126.1	C22—C23—H23	120.1
N4—C9—C8	107.67 (16)	C23—C24—C19	120.7 (2)
N4—C9—C10	121.66 (17)	C23—C24—H24	119.7
C8—C9—C10	130.65 (16)	C19—C24—H24	119.7
			
C2—N1—N2—C4	1.4 (2)	N4—N3—C11—N1	−53.4 (2)
C11—N1—N2—C4	177.87 (16)	C7—N3—C11—N1	122.34 (19)
C7—N3—N4—C9	2.3 (2)	N1—C11—C12—C19	155.84 (17)
C11—N3—N4—C9	178.86 (16)	N3—C11—C12—C19	−22.9 (3)
N2—N1—C2—C3	−0.2 (2)	N1—C11—C12—C13	−24.0 (3)
C11—N1—C2—C3	−176.49 (17)	N3—C11—C12—C13	157.34 (16)
N2—N1—C2—C1	−179.41 (17)	C11—C12—C13—C14	−45.9 (3)
C11—N1—C2—C1	4.3 (3)	C19—C12—C13—C14	134.32 (19)
N1—C2—C3—C4	−1.0 (2)	C11—C12—C13—C18	137.2 (2)
C1—C2—C3—C4	178.15 (19)	C19—C12—C13—C18	−42.6 (2)
N1—N2—C4—C3	−1.9 (2)	C18—C13—C14—C15	0.4 (3)
N1—N2—C4—C5	176.2 (2)	C12—C13—C14—C15	−176.61 (18)
C2—C3—C4—N2	1.8 (2)	C13—C14—C15—C16	−0.8 (3)
C2—C3—C4—C5	−176.1 (2)	C14—C15—C16—C17	0.2 (3)
N4—N3—C7—C8	−1.3 (2)	C15—C16—C17—C18	0.8 (3)
C11—N3—C7—C8	−177.52 (17)	C16—C17—C18—C13	−1.3 (3)
N4—N3—C7—C6	179.22 (17)	C14—C13—C18—C17	0.7 (3)
C11—N3—C7—C6	3.0 (3)	C12—C13—C18—C17	177.66 (18)
N3—C7—C8—C9	−0.2 (2)	C11—C12—C19—C20	−46.6 (2)
C6—C7—C8—C9	179.26 (19)	C13—C12—C19—C20	133.23 (17)
N3—N4—C9—C8	−2.4 (2)	C11—C12—C19—C24	137.91 (19)
N3—N4—C9—C10	175.92 (17)	C13—C12—C19—C24	−42.3 (2)
C7—C8—C9—N4	1.6 (2)	C24—C19—C20—C21	−0.2 (3)
C7—C8—C9—C10	−176.5 (2)	C12—C19—C20—C21	−175.81 (16)
N2—N1—C11—C12	122.87 (19)	C19—C20—C21—C22	−1.3 (3)
C2—N1—C11—C12	−61.3 (3)	C20—C21—C22—C23	1.2 (3)
N2—N1—C11—N3	−58.3 (2)	C21—C22—C23—C24	0.4 (3)
C2—N1—C11—N3	117.47 (19)	C22—C23—C24—C19	−1.9 (3)
N4—N3—C11—C12	125.4 (2)	C20—C19—C24—C23	1.7 (3)
C7—N3—C11—C12	−58.9 (3)	C12—C19—C24—C23	177.30 (16)

### 1,1'-(2,2-Diphenylethene-1,1-diyl)bis(3,5-dimethyl-1H-pyrazol-2-ium) dichloride Hydrogen-bond geometry (Å, º)

*Cg*4 is defined as the centre of gravity of the C19–C24 ring.

**Table:**

*D*—H···*A*	*D*—H	H···*A*	*D*···*A*	*D*—H···*A*
N2—H2*N*···Cl1	0.87 (2)	2.02 (2)	2.8853 (18)	172 (2)
N4—H4*N*···Cl2	0.78 (2)	2.24 (2)	3.0001 (17)	165 (2)
C1—H1*B*···Cl2	0.98	2.74	3.577 (2)	144
C6—H6*C*···Cl1	0.98	2.73	3.614 (2)	150
C8—H8···Cl1^i^	0.95	2.68	3.5352 (19)	149
C10—H10*A*···*Cg*4^ii^	0.98	2.91	3.871 (2)	168
C10—H10*B*···Cl2^iii^	0.98	2.69	3.577 (2)	151
C14—H14···Cl1	0.95	2.99	3.944 (2)	179
C18—H18···Cl2^iv^	0.95	3.05	3.553 (2)	115
C23—H23···Cl1^v^	0.95	2.74	3.602 (2)	151

Symmetry codes: (i) −*x*+2, −*y*+1, −*z*; (ii) *x*+1, *y*, *z*; (iii) −*x*+2, −*y*+1, −*z*+1; (iv) −*x*+1, −*y*+1, −*z*+1; (v) −*x*+1, −*y*+1, −*z*.
